# Endoscope-Assisted Microsurgery in Pediatric Cases With Pineal Region Tumors: A Study of 18 Cases Series

**DOI:** 10.3389/fsurg.2021.641196

**Published:** 2021-08-03

**Authors:** Yuankun Cai, Zhongwei Xiong, Can Xin, Jincao Chen, Kui Liu

**Affiliations:** Department of Neurosurgery, Zhongnan Hospital of Wuhan University, Wuhan University, Wuhan, China

**Keywords:** endoscope-assisted microsurgery, microsurgery, pediatric cases, pineal region tumors, hydrocephalus

## Abstract

**Background:** An endoscope-assisted technique was recently introduced to microsurgery (MS) and may compensate for the disadvantages of MS for deep-seated lesions. This study was performed to identify the effectiveness and safety of endoscopic-assisted microsurgery (EAM) and share our experience of EAM for pediatric cases with pineal region tumors.

**Method:** We retrospectively analyzed the clinical data of consecutive pediatric cases with pineal region tumors treated by EAM or MS from January 2016 to June 2020. These data included the patient population, clinical manifestations, preoperative examination findings, surgical approach, pathological results, and clinical outcomes. The clinical outcomes were analyzed in the EAM group and MS group with a focus on the gross total resection (GTR) rate, postoperative hydrocephalus remission rate, and Karnofsky performance score (KPS). Studies on the surgical management of children with pineal region tumors in the last decade were reviewed.

**Result:** Eighteen children successfully underwent tumor resection via MS (*n* = 8) or EAM (*n* = 10). The children's mean age was 11.4 ± 4.7 years, and the male to female ratio was 7:2. Seventeen patients (94.4%) complicated preoperative hydrocephalus, and 16 (88.9%) presented headache with nausea and/or vomiting. The pathological examination revealed germ cell tumors in 11 (61.1%) patients, neuroepithelial tumors in 4 (22.2%) patients, and a pineoblastoma, arachnoid cyst, and atypical teratoid rhabdoid tumor in 1 (5.6%) patient each. GTR was more commonly achieved in the EAM than MS group (80.0 vs. 50.0%, respectively), and the postoperative hydrocephalus remission rate was higher in the EAM than MS group (87.5 vs. 50.0%, respectively). At a mean follow-up time of 23.6 ± 11.5 weeks, the mean improvement of the KPS 6 months postoperatively was greater in the EAM than MS group (24.0 ± 9.7 vs. 17.5 ± 7.1 points, respectively).

**Conclusion:** EAM combines endoscopic and microsurgical techniques and can be safely and effectively performed to achieve GTR of pineal region tumors in pediatric patients. In children with pineal region tumors who have obstructive hydrocephalus, EAM could improves hydrocephalus remission rates by checking and clearing the midbrain aqueduct under visualization.

## Introduction

Pineal region tumors constitute 2.8 to 11.0% of all intracranial tumors in children, but only <1.0% of brain tumors in adults (1–4). The clinical manifestations of patients with Pineal region tumors are highly variable and usually associated with mass effect in adjacent structures. Although a comprehensive treatment strategy including surgery, radiotherapy, chemotherapy, and a combined strategy has been developed for the treatment of patients with pineal region tumors, surgical resection still generally plays an important role in the management of pineal region tumors because of the need for tissue diagnosis and management of hydrocephalus (5).

However, the difficulty and risk of surgery are considerable because pineal region tumors are deep-seated and surrounded by vital neurovascular structures (6). Additionally, the limited viewing angle of the surgical field makes it difficult to perform total resection and cerebral aqueduct exploration under microscopy, especially for the pediatric patients (7–9). Endoscope-assisted microsurgery (EAM) was recently successfully applied to resection of pineal region tumors (10–13). As an auxiliary tool, the endoscope was used to acquire panoramic visualization of the tumor, helping to compensate for the disadvantages of microsurgery (MS) for pineal region tumors. And, a close observation provided by the endoscope allow meticulous microsurgical manipulation with a minimally invasive technique. EAM has been performed for pediatric patients with pineal region tumors in our institution since January 2018. Based on our previous experience in MS for pediatric patients with pineal region tumors, we conducted a retrospective study to evaluate the safety and efficacy of EAM vs. traditional MS and share our experience of EAM for pediatric patients with pineal region tumors.

## Materials and Methods

### Patient Population

This retrospective study included patients aged <18 years who had been diagnosed with pineal region tumors from January 2016 to January 2020, with surgical treatment and follow-up performed at the Divisions of Neurosurgery in Zhongnan Hospital of Wuhan University. Our research was approved by the Research Ethics Board of Wuhan University (No. 2020044). Informed consent was obtained from people who were authorized to give consent for each pediatric patient included in the study.

The children enrolled in this series from 2016 to 2018 underwent MS, which was performed as the routine option before introduction of endoscopy (4-mm-diameter, 18-cm-length endoscope; Karl Storz GmbH & Co., Tuttlingen, Germany). The children enrolled from January 2018 to January 2020 underwent EAM because the endoscopic-assisted technique was generally performed in our department during this period. The clinical data included the patients' age, sex, presentation, neurological examination findings, neuroimaging findings, tumor size, tumor marker concentrations, surgical findings, pathological results, and Karnofsky performance score (KPS) at admission and at 6 months postoperatively.

### Surgical Procedure

Preoperative computed tomography and MRI were performed to evaluate the characteristics of the tumor and the surrounding anatomy for surgical planning. Almost all patients were placed in a sitting position under general anesthesia with the exception of an infant (No. 17) who was placed in the prone position. The supracerebellar infratentorial approach was used for tumors without lateral expansion that were located primarily below the deep cerebral veins, and the occipital transtentorial approach was used for tumors with significant supratentorial expansion.

In the MS group, the pineal region tumor was removed with a standard microsurgical technique. In the EAM group, when tumor removal was accomplished using a standard microsurgical technique, a 0-/30-degree endoscope was fixed with a mechanical arm and introduced into the surgical field to visualize the blind corners of the microscope. All subsequent surgical procedures were performed under endoscopy. A 0-degree endoscope offers an adequate view at the beginning of the third ventricle exploration and facilitates correct orientation inside the ventricle. Then, the surrounding structures were extensively inspected with a 30-degree telescope including the posterior part of the third ventricle in order to detect tumor remnants and to ensure adequate CSF circulation. When the endoscope detected some residual tumor, it was resected with dedicated angled instruments under 30° visualization. The removal of clots and opening of the cerebral aqueduct were verified endoscopically. Postoperatively, the patients were transferred to the neurological intensive care unit for observation.

### Variable Definitions

The patients' demographic characteristics and clinical information were recorded. The extent of resection was determined by the intraoperative findings or early postoperative MRI. Gross total resection (GTR) was defined as no evidence of a residual tumor, and subtotal resection was defined as <20% residual tumor. The change in the KPS was obtained by subtracting the KPS at admission from the KPS at 6 months postoperatively. The endpoint of analysis for all patients was the date of the last follow-up visit.

### Statistical Analysis

Statistical analysis was performed with SPSS Version 20 (IBM Corp., Armonk, NY, USA). Continuous variables are presented as mean ± standard deviation, and categorical variables are presented as frequency and percent.

## Results

The study cohort comprised 18 children (14 male and 4 female) with a mean age of 11 ± 4.68 years. The principal presenting symptom was headache with nausea and/or vomiting in 16 (89%) patients, and Parinaud's syndrome was present in 7 (39%) children. One patient (No. 12) presented without these symptoms, but had a progressive epilepsy which antiepileptic drugs were ineffective. The mean duration of symptoms prior to presentation was 1 to 288 (mean 25.7 ± 67.7) weeks. Seventeen (94.4%) of 18 children had obstructive hydrocephalus before the surgery, and three patients was carried out ventriculoperitoneal shunt (VPS) placement at other hospital preoperatively because of an acute increased intracranial pressure. The diagnosis of pineal region mass was confirmed by magnetic resonance imaging (MRI) with contrast enhancement before the surgery. There are three children had positive tumor markers in the peripheral blood. The clinical data of cases in our series be summarized in Table 1.

**Table 1:** Summary of cases in our series.

**No**	**Age/sex**	Tumor markers^*^	**Preo-HCP/** **treatment**	**Operation**	**Pathological results**	**Extend of resection**	**Adjuvant therapy**	**Posto-HCP/** **treatment**	**KPS in 6 m-s** **postoperatively**
1	14/M	Normal	+/None	MS	Germinoma	GTR	RT	–	90
2	5/M	Normal	+/None	MS	PBs	STR	CRT	+/VPS	80
3	9/M	Normal	+/VPS	MS	IT	GTR	CRT	–	80
4	7/F	β-HCG↑	+/None	MS	Germinoma	GTR	RT	–	100
5	12/F	Normal	+/None	MS	AA(WHO III)	STR	CRT	+/VPS	90
6	16/M	Normal	+/None	MS	Germinoma	GTR	RT	–	100
7	18/M	Normal	+/VPS	MS	Germinoma	STR	RT	–	80
8	10/F	AFP↑, β-HCG↑	+/None	MS	CC	STR	CRT	+/VPS	80
9	11/M	Normal	+/None	EAM	Germinoma	GTR	RT	–	100
10	8/M	Normal	+/None	EAM	Germinoma	STR	RT	–	90
11	16/M	Normal	+/VPS	EAM	MT	GTR	–	–	90
12	13/F	Normal	–/None	EAM	Arachnoid Cyst	GTR	–	–	100
13	13/M	AFP↑	+/ None	EAM	mGCTs	STR	CRT	–	80
14	14/M	Normal	+/None	EAM	MT	GTR	–	–	100
15	18/M	Normal	+/None	EAM	Ependymoma	GTR	CRT	–	90
16	14/M	Normal	+/None	EAM	DNT	GTR	–	+/VPS	100
17	1/M	Normal	+/None	EAM	ARRT	GTR	CRT	–	100
18	6/M	Normal	+/None	EAM	DA (WHO II)	GTR	CRT	–	90

*Preo-HCP, preoperative hydrocephalus; Posto-HCP, postoperative hydrocephalus; VPS, ventriculoperitoneal shunt; MS, microsurgery; EAM, endoscopic-assist microsurgery; PBs, pineoblastoma; IT, immature teratoma; AA, anaplastic astrocytoma; CC, choriocarcinoma; MT, mature teratoma; mGCTs, mixed germ cell tumor; DNT, dysembryoplastic neuroepithelial tumor; ARRT, atypical teratoid/rhabdoid tumor; DA, diffuse astrocytoma; GTR, gross total resection; STR, subtotal resection; RT, radiotherapy; CRT, chemoradiotherapy; KPS, Karnofsky performance score.*

* *Tumor markers obtained from serum or plasma samples*.

All patients successfully underwent tumor resection by EAM (*n* = 10) or MS (*n* = 8), and no surgical mortality was recorded. The supracerebellar infratentorial approach was used in 17 patients in a sitting position, and the occipital transtentorial approach was performed in one patient. A total of 12 (66.67%) patients achieved GTR, 8 (80%) of 10 patients in the EAM group and in four (50%) of eight patients in the MS group. In the other eight patients, postoperative MRI revealed the presence of residual tumor in the primary area of the tumor, which was defined as a subtotal resection (STR). Except for two patients (NO.5 and NO.8) with transient upgaze paralysis after operation, no serious complications and air embolisms occurred during the perioperative period. Preoperative hydrocephalus was relieved by surgical resection in 10 children, and four other patients underwent shunt surgery due to unrelieved preoperative hydrocephalus.

Pathological examination revealed GCTs in 11 (61.1%) patients, neuroepithelial tumors in four (22.2%) patients, and a pineoblastoma, arachnoid cyst, and atypical teratoid rhabdoid tumor in 1 (5.6%) patient each. All the children with germinomas underwent the postoperative radiotherapy, and eight patients with malignant tumors (including four neuroepithelial tumors, a mixed germ cell tumor, a choriocarcinoma, a pineoblastoma, and an atypical teratoid rhabdoid tumor) underwent postoperative chemoradiotherapy.

During the follow-up (23.6 ± 11.5 months) period, patients were visited once every 3 months. Six months postoperatively, the KPS had significantly improved from that before the treatment total, but the change in the KPS was not as high in the MS group as in the EAM group (17.5 ± 7.1 vs. 24.0 ± 9.7, respectively) (Table 2). Seventeen patients achieved a more consistent status and returned to campus, unfortunately, one patient (No. 2) with a pineoblastoma died of disease progression 30 months postoperatively.

**Table 2:** Comparison of clinical outcomes in MS group and EAM group.

**Variable**	**MS group (*n* = 8)**	**EAM group (*n* = 10)**
Age (mean ± SD, years)	11.4 ± 4.2	11.4 ± 2.8
Tumor size (mean ± SD, cm^2^)	7.8 ± 2.6	6.9 ± 3.8
Remission rate of hydrocephalus under tumor resection (%)	3/6 (50.0%)	7/8 (87.5%)
GTR (%)	4/8 (50.0%)	8/10 (80.0%)
ΔKPS (mean ± SD)	17.5 ± 7.1	24.0 ± 9.7

*MS, microsurgery; EAM, endoscopic-assist microsurgery; GTR, gross total resection; KPS, Karnofsky performance score; ΔKPS, mean improvement of KPS after 6 months postoperatively*.

### Illustrative Case

**Figure 1:**
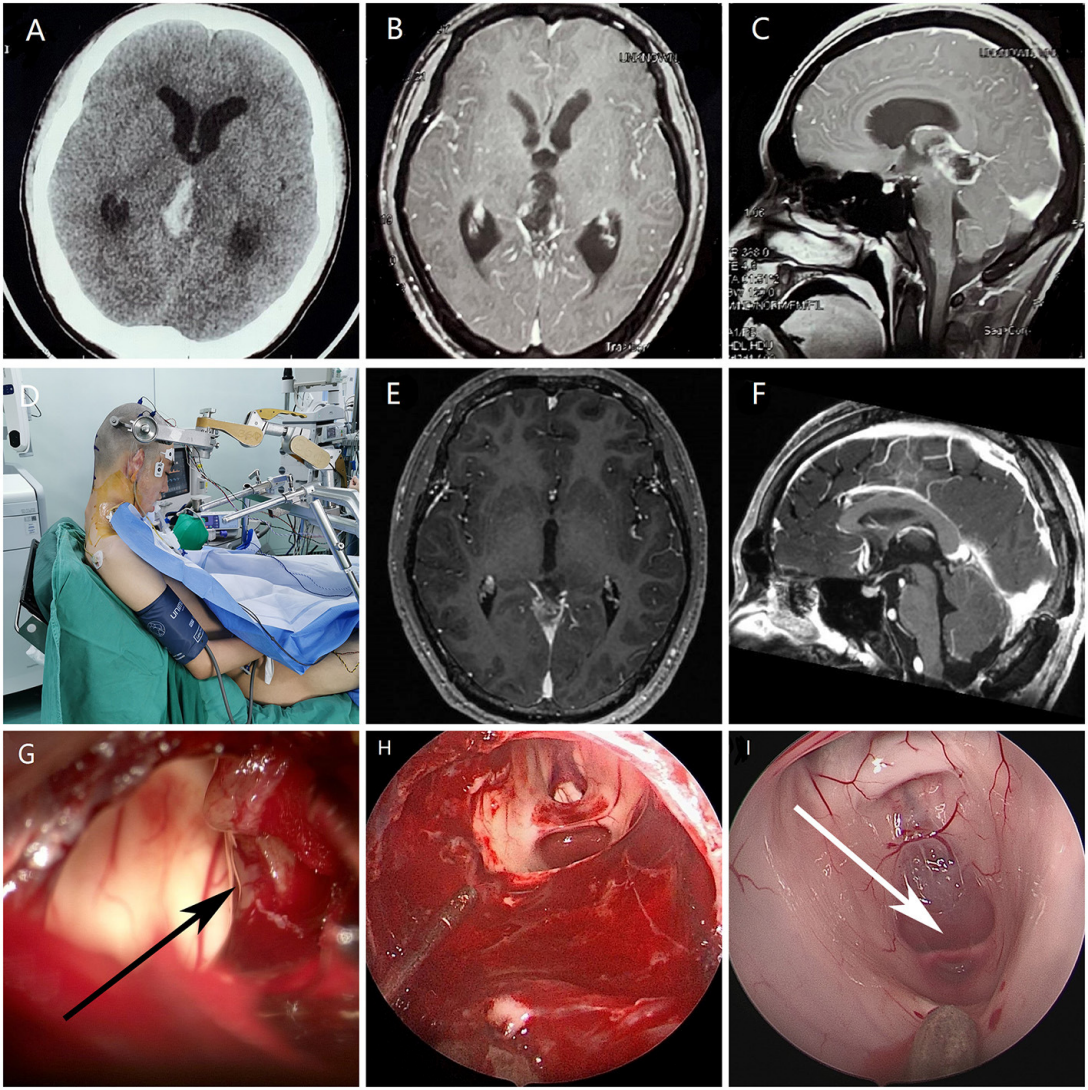
Images of a patient with an ependymoma undergoing EAM. **(A)** Preoperative computed tomography showed a mass in the pineal region with bleeding **(B,C)** Preoperative axial and sagittal magnetic resonance imaging showed a pineal region tumor and obstruction of the cerebral aqueduct. **(D)** Sitting position with the head maximally flexed. **(E,F)** Postoperative magnetic resonance imaging showed complete resection of the tumor and remission of obstructive hydrocephalus at 1 month postoperatively. **(G)** The tumor (black arrow) and bleeding were observed under microscopy. **(H)** Resection of residual tumor by endoscopic surgery. **(I)** Entrance of the aqueduct (white arrow) under endoscopy.

A 12-year-old male patient was admitted to the hospital because of headache and vomiting for 7 days. Brain CT and MRI revealed a large, heterogeneously enhancing mass with bleeding within the pineal gland compressing the tectum and aqueduct of Sylvius, causing obstructive hydrocephalus (Figures 1A–C). He undergone a EAM by supracerebellar-infratentorial approach in sitting position (Figure 1D). First, the tumors and bleeding were found and most of them were cleared under microscope (Figure 1G). Then, the endoscope was introduced into the surgical field, and the residual tumor was resected with dedicated angled instruments under 30° visualization (Figure 1H). The removal of clots and opening of the cerebral aqueduct were verified endoscopically (Figure 1I). The histologic diagnosis was ependymoma in the pineal region. And MRI showed complete resection of the tumor and remission of obstructive hydrocephalus at 1 month postoperatively (Figures 1E,F). The patient had an uneventful postoperative course and received radiotherapy because of the histologic features of the lesion.

## Discussion

Given the pathological diversity found in this region, tissue diagnosis is necessary to determine treatment options, including the choice of adjuvant therapy and estimation of prognosis (5, 14–16). Stereotactic biopsy or neuroendoscopic biopsy may be good choice of obtaining tissue samples, but remains a risk of hemorrhageand the rates of missed or misdiagnosis (5, 17, 18). Another option is tumor resection, which on one hand can obtain more tissue samples to improve diagnostic accuracy; on the other hand, it can also reduce the tumor burden (19, 20). Resection is usually complete and curative for the third of tumors that are benign. There are some evidence favors more radical resection when possible in order to improve the response to adjuvant therapy (5). In addition, patients with mild hydrocephalus gain a particular benefit with open surgery, as total resection may preclude the need for shunting (5, 15). Therefore, tumor resection generally plays an important role in the management of pineal region tumors.

In recent decades, with the application and development of microscopic techniques in neurosurgery, great advances have been achieved in the surgical treatment of the pineal region (21–25). Various surgical approaches have also been developed, which are usually grouped into two categories: supratentorial and infratentorial (15, 26). The choice of surgical access is influenced not only by the surgeon's experience, but also by the location of the deep veins in relation to the tumor. Since the deep veins are often located superior and dorsal to the pineal gland, infratentorial supracerebellar approach is most often used to pineal region tumor resection. The infratentorial supracerebellar approach is optimal for the sitting position (5). Gravity minimizes the pooling of blood in the operative field and facilitates dissection of the tumor from the deep venous system. In our study, 17(94.4%) patients experience tumor resection *via* supracerebellar infratentorial approach in a sitting position. Eleven (64.7%) out of them obtain successfully GTR, and without serious complication (e.g., air embolism, pneumocephalus, and subdural hematoma).

However, microscopes have their inherent disadvantages such as a narrow field of view, shallow depth of field, and limited angle (27). As a result, it is difficult to detect residual tumors and blood clots in the “blind areas” with microscope (6, 9, 20). This is more visible in pediatric patients, as pineal region tumors in children tend to be large and extend in various directions, particularly from the third ventricle to the lateral ventricle (21, 28). The current surgical treatment of pineal region tumors is still challenged by the deep location of these tumors and their close relationship to surrounding vital neurovascular structures. Recent advances in neuroendoscopy have demonstrated that intraoperative use of the endoscope improves visualization of critical structures, especially behind corners that are difficult to visualize optimally using traditional microscopic techniques (6, 13, 20, 29). This seems to bring a new perspective to traditional microsurgery.

EAM is used for procedures in which endoscopy is performed in addition to microsurgical manipulations under the operating microscope during the same operation (13, 30–32). The advantage of EAM is that it combines the strength of microsurgery via microscope and endoscope. Difficult and potentially dangerous manipulations are performed under the microscope. The superior image obtained with the endoscope is exploited for to access the remnant of the tumor, its relative micro-anatomical structures, and the third ventricle. The endoscope aids in visualizing some blind regions thanks to its wider angle of view and its ability to look around corners. Flexible mirrors are used to examine the inferior portion of the tumor bed in order to verify the extent of resection and to avoid leaving any blood clots. Sometimes, simple endoscopic inspection of otherwise inaccessible regions can be used to visualize details that will have major influences on the surgical strategy. The introduction of the endoscope under microscopic control is recommended to decrease the risk of accidental mechanical injury. This has the aim of taking full advantage of both the visualization tools while limiting the interruption of the surgical workflow. In our experience, although adequate exposure for tumor extirpation was obtained under microscopy, the wider visualization provided by the endoscope further confirmed the absence of residual tumor in some cases. In this study, 10 children understand EAM, GTR was achieved in 80% patients in the EAM group, higher than the 50% in the MS group. By bringing light into the surgical field, the surgical microscope can be effectively supported by the optical properties of modern endoscopes.

Approximately 90% of patients with pineal region tumors have hydrocephalus at the time of presentation, due to compression of the third ventricle and cerebral aqueduct by the tumor (7, 8). Therefore, it is essential that hydrocephalus management should be considered in the clinical treatment of pineal region tumors. Microsurgical resection of a tumor in the pineal region involves opening of the occluded aqueduct of Sylvius to resolve the obstructive hydrocephalus, and the postoperative remission rate of hydrocephalus reportedly ranges from 46.7 to 82.1% (5, 20, 33). In our study, the children with obstructive hydrocephalus in EAM first underwent detailed tumor resection under a microscope. Then, the endoscope was used to carefully remove the deepest extensions of the lesions as well as to bring some blind regions (for example, extensions into the quadrigeminal plate and/or the cerebral aqueduct) into the surgical field that may not be adequately and safely visualized with the microscope alone. Checking of the cerebral aqueduct and clearing of residual tumor or blood clots can significantly improve the remission rate of hydrocephalus. Hydrocephalus was relieved more frequently in the EAM than MS group (87.5 vs. 50.0%, respectively). Therefore, EAM was a safe and effective approach for children with pineal region tumors and relieved obstructive hydrocephalus quickly in a one-stage surgery. However, there was still one patient in the EAM group who had to have a shunt implanted because of postoperative hydrocephalus. We speculate that the obstructive hydrocephalus may have been caused by some small blood clots in the lower aqueduct.

The treatment goal for pediatric patients with pineal region tumors is to achieve long-term patient survival and increased quality of life. Our study found that endoscopic-assisted technology can be used as a supplement to MS to achieve a better performance in in tumor resection and hydrocephalus remission. But the study has inherent limitations because of its retrospective nature. The size of the series limited our ability to perform a subgroup analysis aside from patients with pineal region tumors. And, because pineal region tumors are rare, larger sample data are needed to verify them in the future.

## Conclusion

EAM is a safe and effective treatment that achieves a satisfactory GTR rate in pediatric patients with pineal region tumors. For pediatric patients with obstructive hydrocephalus, EAM could improves hydrocephalus remission rates by checking and clearing the midbrain aqueduct under visualization.

## Data Availability Statement

The raw data supporting the conclusions of this article will be made available by the authors, without undue reservation.

## Ethics Statement

The studies involving human participants were reviewed and approved by Research Ethics Board of Wuhan University. Written informed consent to participate in this study was provided by the participants' legal guardian/next of kin. Written informed consent was obtained from the minor(s)' legal guardian/next of kin for the publication of any potentially identifiable images or data included in this article.

## Author Contributions

KL and JC: conception and design. YC, ZX, and CX: acquisition of data and drafting the article. All authors critically revised and reviewed submitted version of manuscript. KL approved the final version of the manuscript on behalf of all authors.

## Conflict of Interest

The authors declare that the research was conducted in the absence of any commercial or financial relationships that could be construed as a potential conflict of interest.

## Publisher's Note

All claims expressed in this article are solely those of the authors and do not necessarily represent those of their affiliated organizations, or those of the publisher, the editors and the reviewers. Any product that may be evaluated in this article, or claim that may be made by its manufacturer, is not guaranteed or endorsed by the publisher.
